# Strengthening international response capacity: International Infectious Diseases Fellowship Programme for Japanese clinicians

**DOI:** 10.5365/wpsar.2024.15.5.1103

**Published:** 2025-10-20

**Authors:** Takuya Adachi, Yayoi Murano

**Affiliations:** a Division of Infectious Diseases, Tokyo Metropolitan Toshima Hospital, Tokyo, Japan.; b Division of Pediatrics, Tokyo Metropolitan Toshima Hospital, Tokyo, Japan.

Established in 1898, Tokyo Metropolitan Toshima Hospital (Toshima Hospital) has a longstanding history of caring for patients with infectious diseases. (*1*) Its communicable disease isolation unit, originally built in the pre-World War II era, was designed to receive patients with smallpox, diphtheria and epidemic typhus. Since then, the unit has continued to lead the treatment of infectious diseases in the Tokyo Metropolitan Region. As public health and sanitation improved, the population’s health needs shifted from infectious diseases to noncommunicable diseases. The COVID-19 pandemic highlighted to hospital management the critical importance of maintaining infectious disease clinicians who are equipped to provide optimal patient care, especially for vulnerable populations such as older adults. Motivated by the rapidly unfolding COVID-19 pandemic and the dedication of clinicians to contribute their expertise to coordinated international outbreak response efforts, Toshima Hospital joined the Global Outbreak Alert and Response Network (GOARN) in 2020.

Established by the World Health Organization (WHO) in 2000, GOARN coordinates the rapid deployment of technical experts worldwide to respond to infectious disease outbreaks and public health emergencies. (*2*) The Network mobilizes professionals from public health institutions, academic centres and other organizations to provide technical support, contain outbreaks and strengthen local response capacities. When a request for assistance is received from a WHO Member State, GOARN partners offer technical expertise through the GOARN Knowledge Platform. (*3*)

A specific criterion to be selected for deployment is that experts must have relevant international work experience and language proficiency. For Japanese clinicians, who typically work at national institutions in small, specialized teams speaking primarily Japanese, acquiring skills such as collaborating in multicultural and multidisciplinary outbreak response teams can be challenging. As a result, many Japanese doctors, despite having strong clinical skills, do not feel prepared to offer their assistance in response to GOARN requests. Clinical trainers discussed how to strengthen these competencies and developed a fellowship programme to support clinicians interested in participating in international deployments, specifically through GOARN. This report describes the key components of the International Infectious Diseases Fellowship Programme, which was launched in 2022.

## ACTION

### Programme development

While core clinical skills can be developed within their institutions, Japanese doctors aspiring to deploy through GOARN also need training in competencies that are best acquired in field settings, such as working in multicultural teams, producing results under pressure and being a first responder in emergencies. The authors held a series of career design workshops for health professionals and students, with presenters from Médecins Sans Frontières (MSF) Japan, the Japanese Red Cross and other nongovernmental organizations (NGOs), during which the workshop participants’ ideal career development was also discussed. (*4*) The fellowship programme was designed in line with the GOARN Competency Model for an international responder. Upon programme completion, fellows are encouraged to pursue further training opportunities, such as that offered by the 3-tiered GOARN Capacity Strengthening and Training Programme. (*5*)

### Aim and objectives

The aim of the fellowship programme is to train clinicians to provide clinical management during public health emergencies in an international setting. At the end of the fellowship programme, clinicians will be able to:

- provide appropriate clinical care in the relevant languages, including history taking, physical examination, diagnosis, treatment, communication and record keeping;
- collaborate effectively as part of a multicultural and multidisciplinary team;
- work adaptively to produce results in dynamic, high-stress conditions with limited resources; and
- report the impact of emergencies and crises on individuals and society.

### Programme description

The 3-year programme consists of three components: clinical training in Japan, international work experience and international study. Fellows engage in clinical practice at Toshima Hospital for a minimum of 6 months per year. For the remaining months, fellows apply for international field deployment through NGOs for up to 6 months as stipulated by MSF. (*6*) Fellows select an NGO to apply to and choose a suitable project and country in which to work. They also apply for international study programmes lasting up to 3 months during the fellowship. For those without international work experience aiming to practice medicine in low- and middle-income countries, beginning study in the first year of the programme is a prerequisite.

### Applicant eligibility criteria

Applicants must:

- hold a Japanese medical licence;
- have completed a 2-year junior residency and a 3-year senior residency training programme;
- be willing to participate in international humanitarian projects; and
- possess a Test of English as a Foreign Language (TOEFL) or International English Language Testing System (IELTS) score that meets the requirements for admission to study programmes abroad.

### Course status

The first fellow, accepted in 2022, earned a 3-month diploma in tropical medicine and hygiene from the Liverpool School of Tropical Medicine, United Kingdom of Great Britain and Northern Ireland, and subsequently participated in field activities in Nepal and Kenya. The second fellow had begun training at the time of writing of this report.

## Discussion

Traditionally, the training of Japanese clinicians has been oriented towards domestic clinical practice, with most doctors completing their education and postgraduate training in national teaching hospitals. While this approach is well suited for preparing clinicians to work within Japan’s health system, it provides limited exposure to the challenges of practicing medicine in international or humanitarian contexts. Few institutions in Japan offer formal training pathways for clinicians who aspire to work in low- and middle-income countries or contribute to international public health emergency responses.

The COVID-19 pandemic reinforced the global nature of infectious disease threats and emphasized the urgent need for a skilled, deployable health emergency workforce. Yet, despite having strong clinical foundations, Japanese clinicians often feel unprepared to respond to international calls for assistance, such as those issued through GOARN, due to gaps in language proficiency, cross-cultural collaboration experience and familiarity with multidisciplinary field-based emergency operations. This context provided the impetus for Toshima Hospital to launch its International Infectious Diseases Fellowship Programme.

The programme represents an innovative effort to integrate international deployment experience into clinicians’ learning journeys and longer-term career development. (*7*) It is designed to complement existing clinical training with practical and varied exposure to global health settings that are aligned with the GOARN Competency Model.

Through a structured pathway that includes domestic service, fieldwork with international organizations and academic study, the fellowship aims to produce well rounded clinicians capable of contributing meaningfully to international outbreak response efforts. However, implementing and sustaining such a programme poses institutional challenges. Toshima Hospital is part of the Tokyo Metropolitan Hospital Organization, which comprises 14 hospitals that compete for limited professional development funding. The future of the fellowship therefore depends on its continued ability to demonstrate value – both to clinicians seeking global health experience and to the hospital system, which must maintain a stable national health workforce.

Successful implementation requires a careful balance between domestic service delivery needs and the aspirations of clinicians to serve internationally. Matching applicants who are motivated to gain international experience with the hospital’s operational requirements is critical. Furthermore, there is a need for ongoing institutional support, flexible staffing models and strong partnerships to ensure sustainability.

### Conclusion

Although still in its early stages, the fellowship programme is a promising model for other institutions in Japan and the Western Pacific Region. It directly addresses the gap between strong domestic clinical public health training and the competencies required for international response work. By preparing clinicians to offer assistance through GOARN and similar mechanisms, the programme contributes to strengthening the global health emergency workforce, a goal increasingly recognized as critical to the regional and global health community.
